# Evaluation of preoperative prediction of intestinal invasion in patients with ovarian cancer

**DOI:** 10.1002/ijgo.13492

**Published:** 2020-12-22

**Authors:** Takashi Takeda, Shigenori Hayashi, Yusuke Kobayashi, Kosuke Tsuji, Shimpei Nagai, Eiichiro Tominaga, Tatsuya Suzuki, Shigeo Okuda, Kouji Banno, Daisuke Aoki

**Affiliations:** ^1^ Department of Obstetrics and Gynecology Keio University School of Medicine Tokyo Japan; ^2^ Department of Radiology Keio University School of Medicine Tokyo Japan

**Keywords:** Barium contrast radiography, Computed tomography, Diagnostic imaging, Intestinal invasion, Magnetic resonance imaging, Ovarian cancer, Preoperative

## Abstract

**Objective:**

To optimize prediction for intestinal invasion of epithelial ovarian cancer. It is important to achieve debulking surgery to improve prognosis in ovarian cancer; intestinal resection is adopted if the cancer is invaded and resectable, but the preoperative evaluation method of intestinal invasion is still controversial.

**Methods:**

Patients (n = 174) who underwent primary debulking surgery for epithelial ovarian cancer were recruited for retrospective study; 28 and 146 patients were classified into the invasion and non‐invasion groups, whether they needed intestinal resection or not. We collected clinical data including evaluation of computed tomography (CT), magnetic resonance imaging (MRI), and barium contrast radiography, and analyzed their accuracy.

**Results:**

The sensitivity and specificity for intestinal invasion were 33.3% and 98.6%, 42.9% and 98.6%, and 66.7% and 93.9% in CT, MRI, and barium contrast radiography, respectively. CT and MRI combined showed a sensitivity of 58.3% and specificity of 96.9%; all three methods combined was the most sensitive combination, showing a sensitivity of 79.2% and specificity of 90.8%.

**Conclusion:**

Combination of CT, MRI, and barium contrast radiography predicts intestinal invasion with the highest sensitivity. These three modalities, however, could not predict all intestinal invasion. Patients should be informed of the possibility of unexpected extensive resection.

## Synopsis

Combination of CT, MRI, and barium contrast radiography give the best prediction of intestinal invasion in ovarian cancer.

## 1 INTRODUCTION

Epithelial ovarian cancer is a leading cause of gynecological cancer‐related death in developed countries. In 2018, 295 414 cases were newly diagnosed, and 184 799 patients died from ovarian cancer according to GLOBOCAN reports.^1^ Most patients with ovarian cancer are diagnosed at an advanced stage, resulting in a worse prognosis. The therapeutic strategy of ovarian cancer is a combination of surgery and chemotherapy, and optimal debulking surgery is the most important factor for improving the outcome.^2^, ^3^, ^4^ Ovarian cancer can spread to the intestine by direct invasion and/or abdominal dissemination. To achieve optimal debulking surgery, intestinal resection is usually applied. The survival impact of a complete cytoreduction with bowel resection is still controversial, but several reports have shown acceptable results for bowel, and especially rectosigmoid, resection.^5^, ^6^, ^7^, ^8^ These procedures are usually conducted by a gastrointestinal surgeon and require sufficient resection. It is imperative to identify those patients with ovarian cancer who would benefit from primary complete debulking surgery and those for whom primary incomplete surgery is inevitable. Latest research investigated the molecular profiles of metastatic bowel lesions of serous ovarian cancer and revealed their usefulness as a therapeutic target; however, it is still important to predict bowel involvement to establish an accurate operative strategy.^9^

We conducted this research to reveal the accuracy of preoperative diagnosis of intestinal invasion and to optimize predictions of intestinal invasion in epithelial ovarian cancer.

## 2 MATERIALS AND METHODS

After approval of the ethics committee (2007–0081), 174 patients who underwent primary debulking surgery for epithelial ovarian cancer from January 2013 to December 2018 were consecutively recruited for the retrospective study with written informed consent. Twenty‐eight patients were classified as the intestinal invasion group, and 146 patients were classified as the non‐invasion group, whether or not they needed intestinal resection in their primary debulking surgery and were pathologically proven to have invasion of the intestine. Primary debulking and staging surgery usually included total hysterectomy, bilateral salpingo‐oophorectomy, pelvic and para‐aortic lymph node dissection, omentectomy, and additional blinded biopsy or biopsy for suspicious lesions with ascites or washing cytology. Intestinal resection for invasive ovarian cancer lesions was performed by general surgeons in our institute. The method of intestinal anastomosis and the establishment of colostomy were left to the surgeon's discretion.

Clinical data (age, body mass index [BMI, calculated as weight in kilograms divided by the square of height in meters], medical history, pathological report) were obtained from clinical records. We typically performed preoperative imaging (usually computed tomography (CT), magnetic resonance imaging (MRI), and barium contrast radiography, and sometimes colonoscopy) before surgery and results were collected from the clinical records. Two independent radiologists and the physician who performed endoscopy diagnosed preoperative imaging (CT, MRI, and radiography) and colonoscopy, respectively. When the imaging results indicated suspected intestinal invasion, it was judged as positive for testing. The criteria of positive imaging of intestinal invasion were as follows: intestinal wall was thickened or penetrated by the ovarian tumor on CT or MRI; intestinal mucosa twitched and did not expand with double contrast in barium contrast radiography; and mucosal protrusion or lesion regardless of performing punch biopsy in colonoscopy. Furthermore, when at least one of the imaging methods indicated intestinal invasion, it was judged as positive in the analysis of combination imaging. When the preoperative imaging revealed that the tumors were bowel invasive with many metastatic lesions, for which complete debulking seems hard to achieve, neoadjuvant chemotherapy was selected and these cases were excluded from the study. Finally, those cases in which complete cytoreduction was promising with and without intestinal resection were included. Intraoperative details and operation data (operation time and blood loss) were also obtained. Complete, optimal, and suboptimal debulking surgery was defined as no residual tumor, residual tumor no more than 1.0 cm, and residual tumor more than 1.0 cm, respectively.

Clinical findings were analyzed using Welch's *t* test for mean data comparison and Fisher's exact test for contingency tables analysis using PRISM 8 (GraphPad Inc., San Diego, CA, USA), with significance defined as *P* < .05.

## 3 RESULTS

Among 174 patients with ovarian cancer, 28 (16.1%) needed intestinal resection in their primary debulking surgery (see Table S1). The degree of intestinal invasion varied from staying in the subserosal layer to penetrating the mucosa in pathological assessments; there was no case of multiple bowel involvement. There was no difference in age and BMI between the two groups. There was a total of 47 (27.0%), 46 (26.4%), and 62 (35.6%) patients with serous, endometrioid, and clear cell carcinoma, respectively. The invasion group had significantly more patients with serous carcinoma, and the non‐invasion group had more patients with endometrioid and clear cell carcinoma (*P* < .001). In addition, significantly fewer women in the invasion group achieved complete surgery (*P* = .002). The invasion group who needed intestinal resection did not include Stage I disease, and all these cases were proven to have intestinal invasion by pathological diagnosis. Considering details of the surgery, compared with the non‐invasion group, the invasion group tended to have longer operation times (mean ± standard deviation: 534.0 ± 113.1 min vs 339.8 ± 80.5 min, *P* < .001), more blood loss (3044.4 ± 1343.5 g vs 1198.1 ± 1119.8 g, *P* < .001), and more needed blood transfusion (26 [92.9%] vs 51 [34.9%], *P* < .001) including autologous and allogeneic blood transfusions.

For the postoperative evaluation, there were two cases of anastomotic leakage in the invasion group, and there was no severe operation‐related morbidity in any other patients. Additionally, it took no additional time to start their adjuvant chemotherapy, regardless of whether patients required intestinal resection (42.1 ± 12.1 days vs 39.5 ± 9.3 days) (Table 1).

**TABLE 1:** Characteristics of epithelial ovarian cancer patients with or without intestinal invasion^a^

Characteristics	Invasion (n = 28)	Non‐invasion (n = 146)	*P* value
Age, year	54.9 ± 14.5	54.1 ± 11.2	.803^b^
Body mass index^c^	19.7 ± 2.4	21.2 ± 4.1	.009^b^
Histological type			
Serous	21 (75.0)	26 (17.8)	<.001^d^, ^e^
Endometrioid	2 (7.1)	44 (30.1)	
Clear	4 (14.3)	58 (39.7)	
Mucinous	0 (0.0)	9 (6.2)	
Others	1 (3.6)	9 (6.2)	
Surgical completeness			
Complete	19 (67.9)	133 (91.1)	.002^e^, ^f^
Optimal	6 (21.4)	7 (4.8)	
Suboptimal	3 (10.7)	6 (4.1)	
Surgical stage			
I	0 (0.0)	76 (63.9)	NA
II	3 (13.6)	10 (8.4)	
III	15 (68.2)	30 (25.2)	
IV	4 (18.2)	3 (2.5)	
Operation time, min	534.0 ± 113.1	339.8 ± 80.5	<.001^b^
Blood loss, g	3044.4 ± 1343.5	1198.1 ± 119.8	<.001^b^
Blood transfusion	26 (92.9)	51 (34.9)	<.001^e^
Postoperative days for adjuvant chemotherapy	42.1 ± 12.1	39.5 ± 9.3	.310^b^

Abbreviation: NA, not applicable.

^a^ Values are given as mean ± SD or as number (percentage).

^b^ Welch's *t* test.

^c^ Body mass index (calculated as weight in kilograms divided by the square of height in meters).

^d^ Serous versus non‐serous.

^e^ Fisher's exact test.

^f^ Complete versus incomplete.

We performed a CT scan and MRI for preoperative evaluation and staging of suspicious ovarian cancer unless contraindicated. Additionally, we performed barium contrast radiography in most patients and colonoscopy in some patients to assess intestinal invasion. Among the 174 partcipants, 173 (invasion: 27, non‐invasion: 146) underwent CT, 174 (invasion: 28, non‐invasion: 146) underwent MRI, 122 (invasion: 24, non‐invasion: 98) underwent barium contrast radiography, and 31 (invasion: 13, non‐invasion: 18) underwent colonoscopy. From these patients, 9/27 (33.3%), 12/28 (42.9%), 16/24 (66.7%), and 5/13 (38.5%) were positive by CT, MRI, barium contrast radiography, and colonoscopy, respectively, in the invasion group; while 2/146 (1.4%), 2/146 (1.4%), 6/98 (6.1%), and 0/18 (0.0%) were positive by CT, MRI, barium contrast radiography, and colonoscopy, respectively, in the non‐invasion group. The sensitivity, specificity, positive predictive value, and negative predictive value of CT, MRI, barium contrast radiography, and colonoscopy are summarized in Table 2. Barium contrast radiography was the most sensitive modality with relatively high specificity, whereas CT and MRI, which are used in almost all preoperative cases, had about a 40% sensitivity with high specificity. Meanwhile, colonoscopy can detect and confirm colorectal invasion by sight and biopsy with 100% specificity but suffers from low sensitivity.

**TABLE 2:** The cross table of each modality for detecting intestinal invasion and their accuracy

Imaging modality	Invasion	Non‐invasion	Sens. (%)	Spec. (%)	PPV (%)	NPV (%)
CT (n = 173)						
Positive	9	2	33.3	98.6	81.8	88.9
Negative	18	144				
MRI (n = 174)						
Positive	12	2	42.9	98.6	85.7	90.0
Negative	16	144				
Barium contrast radiography (n = 122)						
Positive	16	6	66.7	93.9	72.7	92.0
Negative	8	92				
Colonoscopy (n = 31)						
Positive	5	0	38.5	100.0	100.0	69.2
Negative	8	18				

Abbreviations: CT, computed tomography; MRI, magnetic resonaonce imaging; NPV, negative predictive value, PPV, positive predictive value; Sens., sensitivity; Spec., specificity.

We analyzed the combined effects of these modalities. Of the 173 patients that underwent both CT and MRI (27 in the invasion group and 146 in the non‐invasion group), 16 were positive for intestinal invasion in at least one of these modalities from the invasion group and three were positive from the non‐invasion group. The sensitivity, specificity, positive predictive value, and negative predictive value were 59.3%, 97.9%, 84.2%, and 92.9% when CT and MRI were combined, respectively (Table 3). A combination of CT and MRI can detect intestinal invasion with nearly the same sensitivity as barium contrast radiography with high specificity.

**TABLE 3:** Accuracy of CT and MRI combination in patients who underwent both modalities (n = 173)

Imaging modality	Invasion (n = 27)	Non‐invasion (n = 146)	Sens. (%)	Spec. (%)	PPV (%)	NPV (%)
CT and MRI						
Positive for at least one modality	16	3	59.3	97.9	84.2	92.9
Negative for both modalities	11	143				

Abbreviations: CT, computed tomography; MRI, magnetic resonaonce imaging; NPV, negative predictive value, PPV, positive predictive value; Sens., sensitivity; Spec., specificity.

We analyzed the additional effect of barium contrast radiography testing combined with CT and MRI. In total, 122 patients underwent all three diagnostic tests: CT, MRI and barium contrast radiography. Barium contrast radiography predicted five more instances of intestinal invasion but added six more instances of false positives. The sensitivity, specificity, positive predictive value, and negative predictive value were 79.2%, 90.8%, 67.9%, and 94.7%, respectively, when using a combination of CT, MRI, and barium contrast radiography (Table 4). There were five patients in whom we could not detect the intestinal invasion preoperatively even when using all three modalities. These patients all showed exclusion from the intestine by barium contrast radiography; but it was difficult to detect invasion from these three modalities (see Table S2).

**TABLE 4:** Accuracy of CT, MRI, and barium contrast radiography combination in patients who underwent these three modalities (n = 122)

Imaging modality	Invasion (n = 24)	Non‐invasion (n = 98)	Sens. (%)	Spec. (%)	PPV (%)	NPV (%)
CT and MRI						
Positive for at least one modality	14	3	58.3	96.9	82.4	90.5
Negative for both modalities	10	95				
CT, MRI and barium contrast radiography						
Positive for at least one modality	19	9	79.2	90.8	67.9	94.7
Negative for all modalities	5	89				

Abbreviations: CT, computed tomography; MRI, magnetic resonaonce imaging; NPV, negative predictive value, PPV, positive predictive value; Sens., sensitivity; Spec., specificity.

**FIGURE 1:**
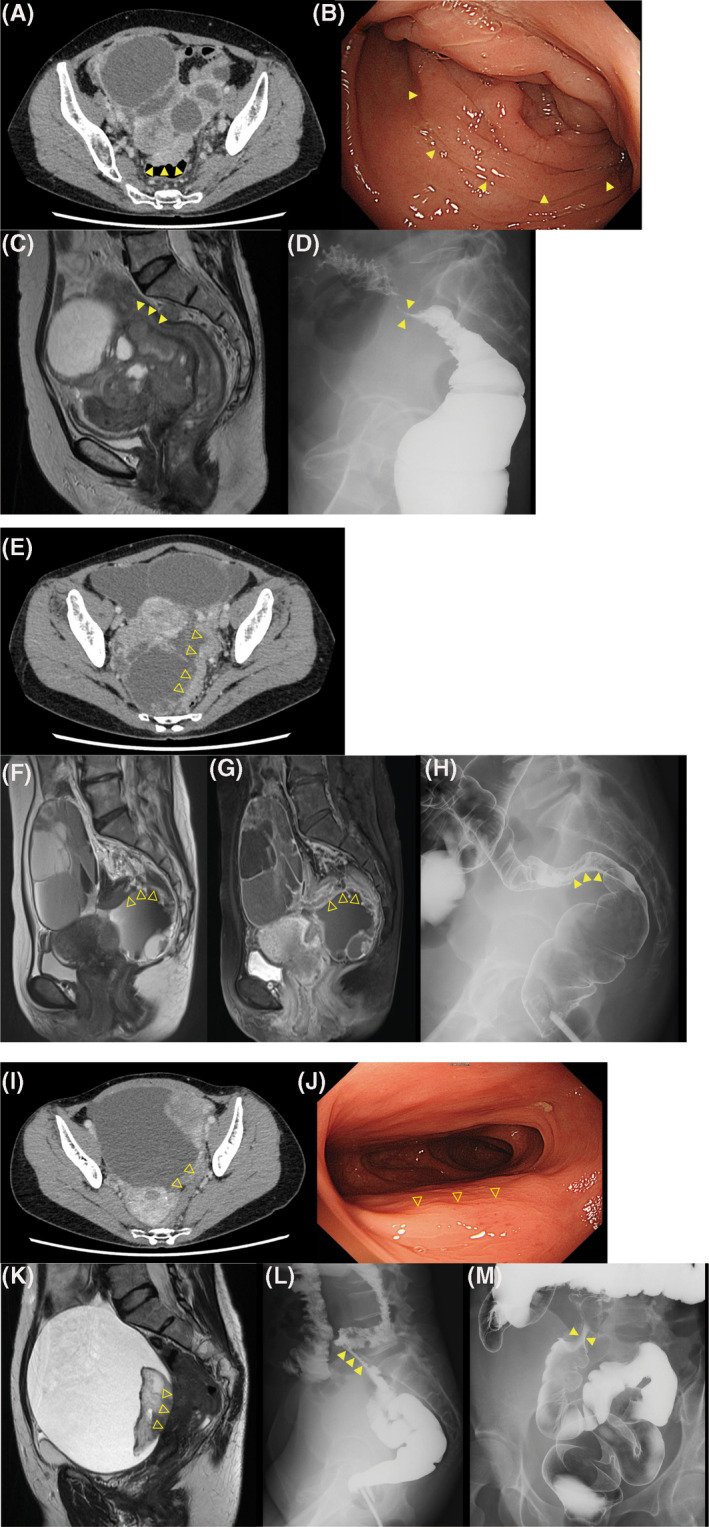
(a–d) Preoperative imaging evaluation of case 1. Patient with ovarian serous carcinoma that needed colorectal resection and pathologically proven to have invasion. In this patient, computed tomography (CT), magnetic resonance imaging (MRI), barium contrast radiography, and colonoscopy all showed invasive findings. (a) Enhanced CT, which indicated a rectal invasion by thickening of the rectum wall (arrowhead). (b) Colonoscopy revealed thickened and edematous mucosa of the rectum with stenosis indicating invasion of carcinoma. (c) MRI showed the interruption of the rectal wall. (d) Barium contrast radiography showed severe stenosis with extension in the anal side of the rectum. (e–h) Preoperative imaging evaluation of case 2. Patient with ovarian serous carcinoma that needed colorectal resection and pathologically proven to have invasion. In this patient, only barium contrast radiography showed positive finding of invasion e, CT showed that rectum came close to the ovarian tumor; the invasiveness of the cancer is not clear. (f) (T2‐weighted) and G (enhanced T1‐weighted) MRI, it was difficult to detect direct invasion to other organs (arrowheads). (h) Barium contrast radiography showed extension from outside of the rectum. Insufflation revealed strong twitching of mucosa, which indicates the intestinal invasion. (i–m) Preoperative imaging evaluation of case 3. Patient with ovarian clear cell carcinoma and operatively free of intestinal invasion but positive for barium contrast radiography (false positive). (i) CT showed retraction of sigmoid colon with some space between the tumor (arrowheads). (j) Colonoscopy showed extra‐intestinal oppression with no evidence of invasion (arrowheads). (k) MRI showed a large ovarian tumor. However, there was no invasive sign to the rectosigmoid colon. l (side view) and m, (front view), barium contrast radiography indicated the rectosigmoid invasion by strong stenosis (arrowheads)

We also investigated the additional effect of adding colonoscopy to CT and MRI diagnosis. Five patients were positive for colonoscopy with pathological confirmation (Table 2). However, they were all diagnosed as having intestinal invasion by CT or MRI, indicating that adding colonoscopy had less effect on the diagnosis of colorectal invasions. Representative sample cases are shown in Figure 1 (a–d patient with invasion to the rectum and all positive results in CT, MRI, barium contrast radiography, and colonoscopy; e–h, patient who had invasion to rectum with positive barium contrast radiography but negative result in CT and MRI; and i–m patient without intestinal invasion but positive for barium contrast radiography).

## 4 DISCUSSION

This research revealed that a combination of CT, MRI, and barium contrast radiography could provide the highest sensitivity (79.2%) with relatively high specificity (90.8%) to predict intestinal invasion by ovarian cancer. CT and MRI, the most popular combination, showed high specificity (96.9%). However, their sensitivity (58.3%) was relatively low compared with barium contrast radiography. On the other hand, detecting intestinal invasion by colonoscopy seemed challenging, but the method could perfectly detect invasion by biopsy and pathological assessment.

It is still important to perform primary debulking and staging laparotomy and achieve complete debulking surgery in ovarian cancer, even if several effective drugs like bevacizumab and olaparib have been introduced. Laparoscopic assessment before debulking surgery may prove a helpful diagnostic tool to aid in the accuracy of clinical and radiological prediction of the optimal debulking surgery.^10^ However, a preoperative assessment of tumor resectability would still be valuable. Recently, a systematic review showed that adding fluorodeoxyglucose‐positron emission tomography and MRI provides high specificity and moderate sensitivity to assess macroscopic incomplete debulking in advanced ovarian cancer.^11^ Several reports have suggested the efficacy of platelet count and other clinical data like BMI, ascites, or carcinomatosis combined with CT, MRI, and/or ultrasound in predicting resectability of advanced ovarian cancer.^12^, ^13^, ^14^, ^15^

The best modality to predict advanced ovarian cancer remains controversial, especially regarding intestinal invasion. Furthermore, it is notable and instructive that even the three combined modalities of CT, MRI, and barium contrast radiography, while appearing to be useful in combination, could not predict all intestinal invasion. As a result, patients who were suspected of ovarian cancer should be informed of the possibility of unexpected extensive resection before their primary debulking surgery.

The availability of barium contrast radiography compared with ultrasonography, CT, and MRI was previously reported, particularly in the diagnosis of deeply infiltrating intestinal endometriosis.^16^, ^17^, ^18^, ^19^, ^20^ CT colonography, a combined technique with CT, became popular and is reported to be a useful diagnostic tool for this type of endometriosis.^21^, ^22^, ^23^ However, it is not yet widely recognized in the field of gynecologic oncology and it is still difficult to correctly diagnose the colorectal invasion of ovarian cancer.^24^ Our results showed that barium contrast radiography may have an additiive effect for detecting colorectal invasion with CT and MRI. However, CT and MRI had relatively useful accuracy. CT colonography may be a promising modality to evaluate tumor resectability for ovarian cancer but there is only one report that investigated the efficacy of CT colonography to detect rectosigmoid involvement in ovarian cancer patients.^25^ Hence, further evaluation will be needed.

The limitation of this study is the small sample size of the intestinal invasion group and the small numbers for colonoscopy. Furthermore, the lack of central and blinded imaging diagnosis, especially the bias that each radiographic interpretation may impact on the diagnosis of subsequent diagnoses, is a limitation.

In conclusion, we showed that a combination of CT, MRI, and barium contrast radiography predicts intestinal invasion for ovarian cancer with the highest sensitivity and acceptable specificity. However, further research of other diagnostic tools is necessary to achieve better accuracy for predicting intestinal invasion of ovarian cancer.

## CONFLICTS OF INTEREST

The authors have no conflicts of interest.

## AUTHOR CONTRIBUTIONS

The study was designed by TT; planned by TT, YK, KT, and KB; and conducted by TT, SH, YK, KT, SN, ET, KB, and DA. Data analysis was by TT, SH, TS, and SO; and the manuscript was written by TT and SH.

## Supporting information

Table S1‐S2
