# Supplementary material for: Evaluation of preoperative prediction of intestinal invasion in patients with ovarian cancer
Source: Int J Gynaecol Obstet. 2020 Dec 22;153(3):398–404. doi: 10.1002/ijgo.13492 (PMC8246869; doi:10.1002/ijgo.13492)
Supplement: Supplementary file 1 — Table S1‐S2 [file IJGO-153-398-s001.docx]

**Supplemental table**

Supplemental table 1. Operation procedures which were performed in intestinal invasion group

| Operation procedure in invasion group (n=28) | |
| --- | --- |
| Low anterior resection | 15 |
| Sigmoid colectomy | 5 |
| Colectomy (except sigmoid) | 4 |
| Small bowel partial resection | 5 |
| Colostomy | 8 |
| (including duplicate) | |

Supplemental table 2. Five patients who could not be detected the intestinal invasion by CT, MRI and barium contrast radiography

|  |  |  |  |  |  |  |  |  |
| --- | --- | --- | --- | --- | --- | --- | --- | --- |
| Case | Age | CT & MRI | Barium contrast radiography | Operation procedure | Acheivement | Pathology | Depth of invasion to intestine | FIGO stage |
| 1 | 53 | no intestinal invasion | Exclusion of sigmoid,  but not invasive | ATH+BSO+OMT+PLN+PAN +Low anterial resection | complete | high grade serous carcinoma | subseosa | IIC |
| 2 | 44 | no intestinal invasion | Exclusion of sigmoid,  but not invasive | ATH+BSO+OMT+PLNBx +high anterior resection | complete | endometrioid carcinoma grade 2 | invasive  (unclear, but not deep) | IIC |
| 3 | 44 | no intestinal invasion | Exclusion of sigmoid,  but not invasive | ATH+BSO+pOMT+PLN+PAN +colectomy+partial hepatectomy | complete | high grade serous carcinoma | subserosa | IIIC |
| 4 | 51 | no intestinal invasion | Exclusion of sigmoid,  but not invasive | ATH+BSO+pOMT+PLNBx +Sigmoidectomy＋colostomy | suboptimal | high grade serous carcinoma | invasive  (unclear, but not deep) | IIIC |
| 5 | 43 | no intestinal invasion | Exclusion of sigmoid,  but not invasive | ATH+BSO+OMT+PLN+PAN +Low anterial resection | optimal | clear cell carcinoma | invasive  (unclear, but not deep) | IIIB |
| ATH: abdominal total hysterectomy, BSO: bilateral salpingo-oophorectomy, OMT: omentectomy, | | | | | | | | |
| PLN: pelvic lymphadenectomy, PAN: para-aortic lymphadenectomy, PLNBx: pelvic lymph node biopsy | | | | | | | | |
